# Analytical data on three Martian simulants

**DOI:** 10.1016/j.dib.2024.111099

**Published:** 2024-11-08

**Authors:** Nicole Costa, Alessandro Bonetto, Patrizia Ferretti, Bruno Casarotto, Matteo Massironi, Francesca Altieri, Jacopo Nava, Marco Favero

**Affiliations:** a Dipartimento di Geoscienze, Università degli Studi di Padova, Via Gradenigo 6, 35131 Padova, (PD), Italy; b Dipartimento di Scienze Ambientali, Informatica e Statistica, Università Ca’ Foscari, Via Torino, 155, 30172 Mestre, Venezia, Italy; c Centro di Ateneodi Studi e Attività Spaziali ``Giuseppe Colombo'' (CISAS), Università degli Studi di Padova, Via Venezia 15, 35131 Padova, (PD), Italy; d Istituto di Astrofisica e Planetologia Spaziali (IAPS), Istituto Nazionale di Astrofisica (INAF), Via Fosso del Cavaliere, 100, 00133 Roma, Italy

**Keywords:** MARS, Mass-spectrometer, XR-diffraction, SEM, Grainsize, Hyperspectral, VNIR, SWIR

## Abstract

The preparation of planetary missions as well as the analysis of their data require a wide use of planetary simulants. They are very important for both testing mission operations and payloads, and for interpreting remote sensing data. In this work, a detailed analysis of three commercially available simulants of Martian dust and regolith is presented. Indeed, up to date, a complete data set related to their chemical, mineralogical, granulometric and spectral characters is not fully provided by their distribution and sales companies. Our dataset regards the Mars Global (MGS-1) High-Fidelity Martian Dirt Simulant [1], the Mojave Mars Simulant MMS-1 [2] and the Enhanced Mars Simulant (MMS-2) [2]. Being essential for ensuring consistency and enabling data comparison, all the chosen Martian simulants underwent the same analytical process. Grainsize data were collected using a Laser Diffraction Particle Size Analyzer. Chemical analysis was performed by Inductively Coupled Plasma Mass Spectroscopy (ICP-MS). Mineralogical analysis was carried out by X-Ray powder Diffractometry (XRD). Moreover, the largest particles of MGS-1 simulant were analyzed with the Scanning Electron Microscope (SEM-EDS) in order to confirm their chemical composition. Finally, the spectral acquisitions in the VNIR-SWIR range were taken by two Headwall Photonics hyperspectral imaging cameras. This complete series of data integrating pre-existing ones (e.g., Cannon et al. [1] and Karl et al. [2]) can in the future be used to allow a straightful choice of the right simulant for biological and life-support experiments and potential testing of mission instruments, to help inferring the composition of the Martian surface from remote sensing data, and to create new simulants or adjust the existing ones in order to get closer to the known Martian regolith variability and eventually new compositional information provided by future missions.

Specifications Table

**Table:** 

Subject	Space and Planetary Science
Specific subject area	Planetary geology; Mars geology; Mars soil simulants
Type of data	Tables, Images, Graphs .docx file Raw, analyzed
Data collection	This dataset contains data derived from chemical, mineralogical, granulometric and hyperspectral acquisitions of Mars Global (MGS-1) High-Fidelity Martian Dirt Simulant [1], Mojave Mars Simulant MMS-1 [2,3] and Enhanced Mars Simulant (MMS-2) [2,4]. The instruments used for this work are: - Laser Diffraction Particle Size Analyzer Malvern Panalytical Mastersizer3000: granulometric analysis. - Inductively Coupled Plasma Mass Spectrometer (ICP-MS) Perkin-Elmer NexION 350X: chemical analysis. - X-Ray powder Diffractometer (XRD) Philips X'Pert PRO: mineralogical analysis. - Scanning Electron Microscope (SEM-EDS) Tescan SOLARIS equipped with Oxford Instruments microanalytical system: chemical analysis. - Headwall Photonics Nano-Hyperspec (400–1000 nm) and Micro-Hyperspec (900–2500 nm) cameras: reflectance measurements.
Data source location	Dipartimento di Geoscienze, Università degli Studi di Padova, Padova, Italia (45°24′33.61″ N; 11°53′37.30″ E)
Data accessibility	Repository name: MartianSimulants Data identification number: 10.25430/researchdata.cab.unipd.it.00001279 Direct URL to data: https://researchdata.cab.unipd.it/id/eprint/1279

## 1 Value of the Data

• Our analytical dataset provides new granulometric, chemical, mineralogical and hyperspectral data of three commercially available simulants of Martian regolith carried out using the same instruments and procedures. This approach ensures consistency of data and allows for better comparison of the different variables.
• The dataset constitutes a deep characterization of Martian regolith and dust, useful for any studies aimed to unravel whether Mars soils can support plant growth by means of microbiological and plant cultivation experiments in Mars analogue environments.
• These data could be used for any interpretation of the Mars surface composition from orbital acquisition through a comparison with reference samples of well-known chemical, mineralogical and spectral properties.

## 2 Background

Planetary simulants are of paramount importance as reference material to infer planetary surface composition from orbital remote sensing data, biological and life-support experiments, testing planetary missions’ payloads, setting up planetary analogue facilities for in situ mission operations. Hence their physical properties as well as their mineralogical and chemical composition must be fully constrained. Nonetheless the commercially available simulants are often accompanied by partial descriptions, acceptable for some applications but not sufficient when detailed characterizations are needed to understand planetary surface compositions. In addition, different sales and distribution companies characterize their samples using different instruments and approaches and thus making less straightforward their comparison. Finally, slight variability of compositional properties of commercially available samples have been documented (e.g., [6]), hence a periodic full characterization is extremely beneficial even considering the continuous technological improvement of the laboratory devices and their analytical capabilities. In this work we have characterized different global and commercially available simulants of the Martian regolith and atmospheric dust. They are indeed particularly relevant for life support experiments in analogue environments and the understanding, from remote sensing data, of the variable dust cover on the Martian surface as well as of the content, grain size and composition of dust-ice intermixing in its polar caps.

## 3 Data Description

This article collects an analytical dataset of the following commercial simulants: the Mars Global High-Fidelity Martian Dirt Simulant (MGS-1), reproducing the average composition of the Red Planet [1], the Mojave Mars Simulant (MMS-1) [3] and the Enhanced Mojave Mars Regolith Simulant (MMS-2) [4] both simulating the regolith composition found by the lander Phoenix which landed at the edge of the North Polar Cap, in the region named Green Valley [5]. However, MMS-1 comes from altered pyroclasts of a cinder cone close to MMS site but not from the original Saddleback basalt of MMS [2,5]. MMS-2 derives from the MMS-1 simulant enhanced with iron and magnesium oxides, silica sand and gypsum to mimic the Opportunity soil measurements [5] and pretending to be representative of the average Martian surface. The bulk composition of the three simulants provided by the sales companies is reported in the following Table 1 and compared with the Martian average composition obtained through NASA Curiosity rover in Gale Crater (Rocknest soil [1,7]) and through NASA lander Phoenix in Green Valley [2].

**Table 1:** Table of elemental composition in weight percentage (wt%) of the chosen simulants. The oxides content of the Martian simulants is compared with oxide content taken in specific reference sites on Mars for each simulant.

Oxide (wt%)	Mars average	MGS-1	Mars average	MMS-1	MMS-2
*Reference*	[1,7]	[8]	[9]	[9]	[9]
SiO_2_	42.97	43.90	43.52	49.40	43.8
TiO_2_	1.19	0.46	0.78	1.09	0.83
Al_2_O_3_	9.37	12.84	8.64	17.10	13.07
Cr_2_O_3_	0.49	–	0.37	0.05	0.04
Fe_2_O_3_	19.18	10.60	18.28	10.87	18.37
MnO	0.42	0.11	0.32	0.17	0.13
MgO	8.69	14.81	6.54	6.08	6.66
CaO	7.26	7.91	6.09	10.45	7.98
Na_2_O	2.70	1.49	2.57	3.28	2.51
K_2_O	0.49	0.29	0.35	0.48	0.37
P_2_O_5_	0.95	0.17	0.79	0.17	0.13
SO_3_	5.47	–	6.42	0.10	6.11

It is worth comparing this data-set with our results which represent a substantial integration of the pre-existing data-set. Indeed, in the following sections together with the chemical data obtained through Inductively Coupled Plasma Mass Spectrosmetry we provide granulometric information using a Laser Diffraction Particle Sizer Analyzer, the mineralogic characterization through X-ray Diffraction and the hyperspectral signatures obtained using hyperspectral imaging cameras.

The dataset of our analysis is contained in the linked repository in Research Data Unipd [10] and it is composed of a single folder named “MartianSimulants”. The folder includes five sub-folders, each one for every instrument used for the characterization. The sub-folders are designated with the instrument name: 1) “granulometer” for the particle size analyzer; 2) “ICP-MS” for the ICP-mass spectrometer; 3) “powderXRD” for the X-ray diffractometer; 4) “SEM-EDS” for the scanning electron microscope; 5) “hyperspectralcameras” for the cameras. In each sub-folder, there are three .pfd files named like the simulants: 1) MGS-1; 2) MMS-1; 3) MMS-2.

**Fig. 1:**
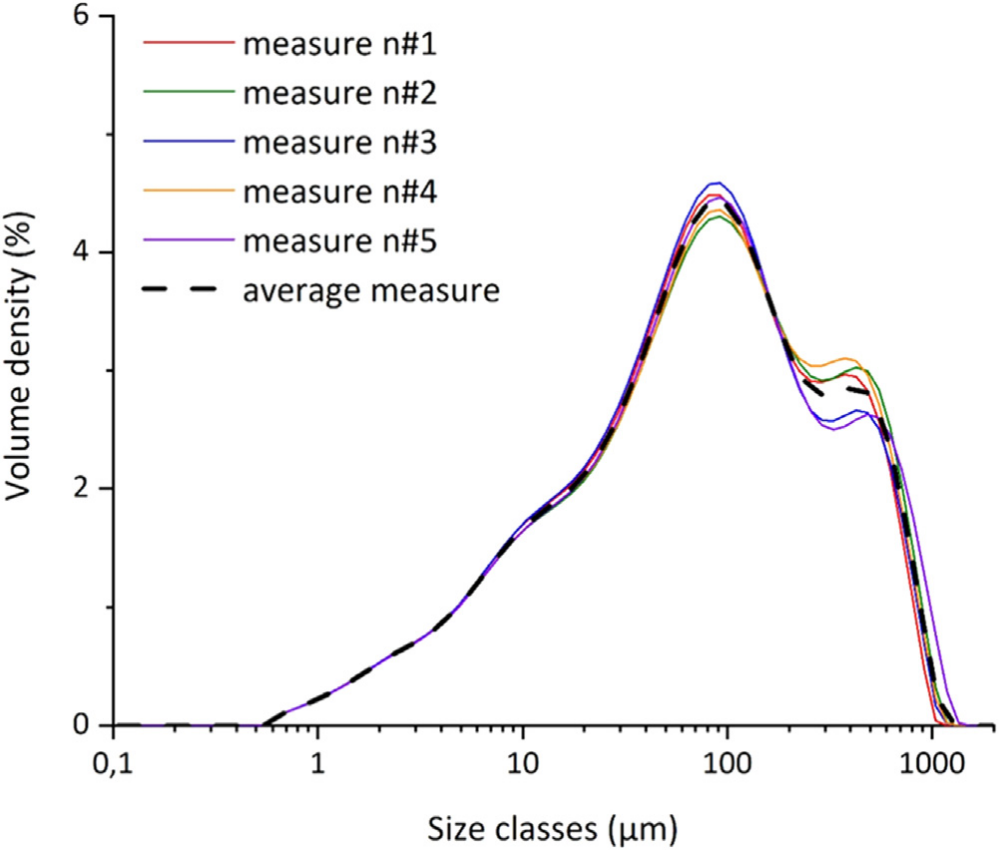
Granulometric curves of the original MGS-1.

In particular, the sub-folder “granulometer” files are named like the simulant: “MGS-1.docx”, “MMS-1.docx” and “MMS-2.docx”. The .docx file includes graph of the grainsizes (Fig. 1) and the related table (Table 2). The diagram in Fig. 1 highlights the grainsize distribution of five acquisitions and their average for the original sample and five different granulometric classes, in which the simulant was sieved (0–32 µm, 32–63 µm, 63–250 µm, 250–1000 µm and >1000 µm). With the term Original sample, we refer to the simulant powder as it was received in the original package. The table reports the five-acquisition data and their average for every class and for the original sample (Table 2).

**Table 2:** Extract of the table derived from granulometric analysis of the original MGS-1.

Size classes (µm)	Measure n#1	Measure n#2	Measure n#3	Measure n#4	Measure n#5	Average measure
0,1061	0,000	0,000	0,000	0,000	0,000	0,000
0,1205	0,000	0,000	0,000	0,000	0,000	0,000
0,1369	0,000	0,000	0,000	0,000	0,000	0,000
0,1556	0,000	0,000	0,000	0,000	0,000	0,000
0,1768	0,000	0,000	0,000	0,000	0,000	0,000
0,2008	0,000	0,000	0,000	0,000	0,000	0,000
0,2282	0,000	0,000	0,000	0,000	0,000	0,000
0,2593	0,000	0,000	0,000	0,000	0,000	0,000
0,2946	0,000	0,000	0,000	0,000	0,000	0,000
0,3347	0,000	0,000	0,000	0,000	0,000	0,000
0,3802	0,000	0,000	0,000	0,000	0,000	0,000
0,4320	0,000	0,000	0,000	0,000	0,000	0,000
0,4908	0,000	0,000	0,000	0,000	0,000	0,000
0,5577	0,008	0,008	0,008	0,008	0,008	0,008
0,6336	0,083	0,084	0,082	0,083	0,082	0,083
0,7199	0,125	0,124	0,124	0,123	0,123	0,124
0,8179	0,162	0,159	0,161	0,158	0,158	0,160

In the sub-folder “ICP-MS,” the file “MGS-1, MMS-1, MMS-2.docx” contains the concentrations of the major and minor elements measured in the three Martian simulants (expressed in mg/g for major elements and µg/g for minor elements) together with their relative standard deviation in percent (RSD%; Table 3). Note that Cadmium-111 concentration was not provided because under the detection limit of the instrument.

**Table 3:** Concentrations of chemical elements and their relative standard deviations in percent (RSD%) for MGS-1, MMS-1 and MMS-2.

Units	mg/gr								µg/gr												
Chemical element		Al 27	Na 23	Mg 24	Ti 47	K 39	Ca 43	Fe 57	Cr 52	V 51	Co 59	Ni 60	Mn 55	Sr 88	Cd 111	Cu 63	Zn 68	Ba 137	Be 9	Pb 208	Tl 205
**MGS-1**	value	23	988	46	3,8	5,5	49	88	747	90	47	557	852	245	N.D.	14	131	154	0,35	0,29	0,41
	RSD%	4,1	3,5	4,4	0,9	1,6	7,3	0,4	6,0	0,7	3,4	1,0	2,0	2,7		17,8	30,2	4,6	10,3	95,6	3,7
**MMS-1**	value	78	1920	20	5,4	20	48	55	113	72	23	74	954	304	N.D.	31	155	622	1,6	7,9	0,6
	RSD%	1,1	1,0	1,8	4,3	0,9	9,9	1,1	2,1	2,9	5,0	2,0	1,9	2,3		2,8	7,5	1,8	16,5	2,1	6,8
**MMS-2**	value	64	1535	28	4,3	17	46	95	88	56	19	64	1149	319	N.D.	38	120	554	1,4	7,7	0,5
	RSD%	1,4	1,3	0,4	2,8	1,2	12,3	0,2	4,9	1,6	4,3	1,6	1,6	1,7		1,4	22,7	1,0	13,6	4,9	15,0

**Fig. 2:**
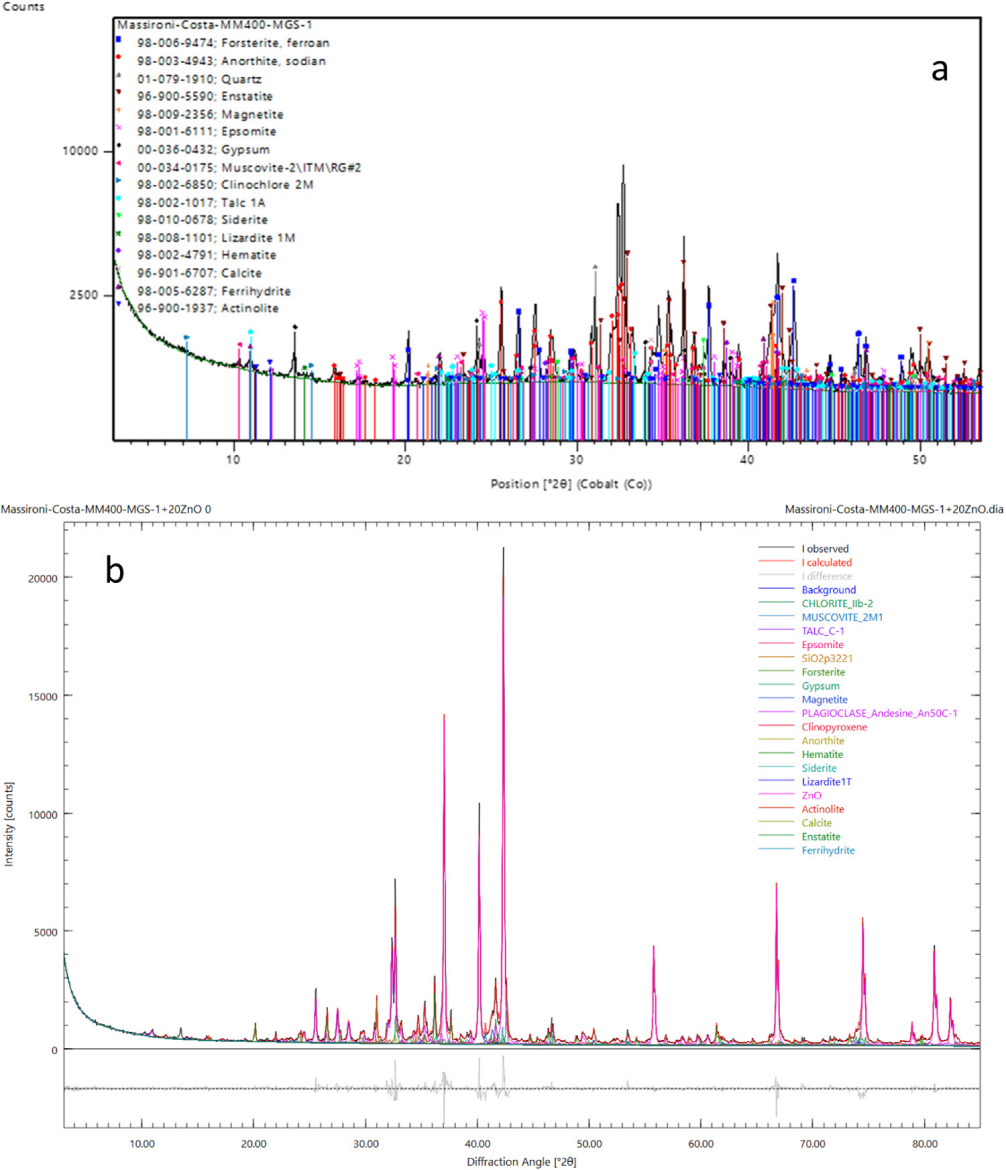
a) XRD pattern related to qualitative analysis of the simulant MGS-1; b) XRD pattern related to quantitative analysis of the simulant MGS-1: minerals detected are shown in different colors. The gray curve at the bottom represents the differences, in terms of intensities, between the measured (black) pattern and the fitted (red) pattern, it shows effectively the goodness of the fit.

The three files in the sub-folder “powderXRD” are named with the simulant names (e.g., “MGS-1.docx”). In each file there are two diffractograms and related tables: the first one is related to a qualitative analysis excluding the amorphous material (Fig. 2a and Table 4a), the second one to a quantitative analysis which however includes the amorphous material (Fig. 2b and Table 4b). Therefore, the first table includes the minerals revealed in the sample (Table 4a), while in the second table the identified minerals are accompanied with their amount in percent (%) (Table 4b). Peaks shown in the diffractograms are linked to specific minerals (Fig. 2a and b). In the database, we also added the diffraction patterns in numerical format.

**Table 4:** a) Table of qualitative analysis of the simulant MGS-1; b) Table of quantitative analysis in mass percentage (in m%) of the simulant MGS-1.

A Components	
Olivine	
Plagioclase	
Quartz	
Pyroxene	
Magnetite	
Epsomite	
Gypsum	
Mica	
Chlorite	
Talc	
siderite	
serpentine	
hematite	
calcite	
ferrihydrite	
amphibole	


B Components	Amount (m%)
olivine	12.6
plagioclase	30.1
quartz	1.7
pyroxene	15.8
magnetite	2.1
epsomite	2.2
gypsum	2.9
mica	2.1
chlorite	1.7
talc	3.0
siderite	0.3
serpentine	0.8
hematite	0.7
calcite	0.3
ferrihydrite	4.6
amphibole	1.9
amorphous	17.2

**Fig. 3:**
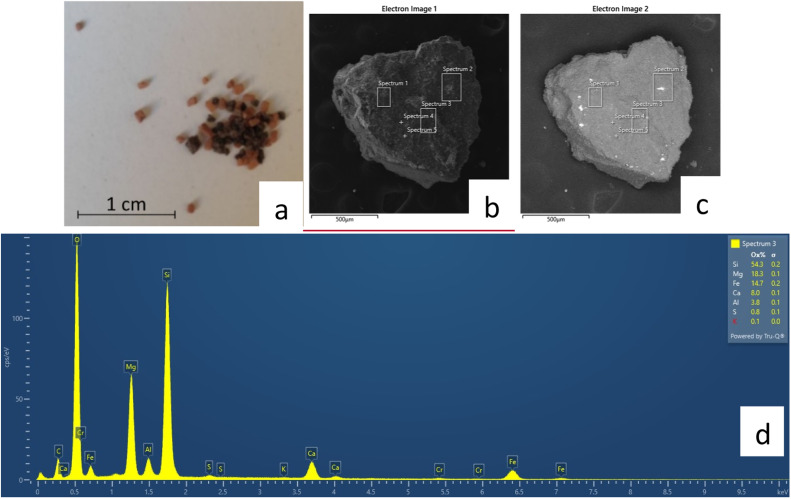
Mineralogical and chemical characterization of the coarser grains of the MGS-1 simulant. a) Pyroxene and gypsum/Al-oxides crystals, corresponding to dark and red samples respectively; b) Secondary Electrons (SE) pictures of the crystal and the sites where the data were collected; c) Back Scattered Electrons (BSE) pictures of the crystal and the sites where data were collected; d) spectrum acquired on the black crystal and related elemental ratio from the energy-dispersive spectroscopy analysis expressed in oxide percentage.

In the sub-folder “SEM-EDS” the file “MGS-1.docx” shows the chemical composition of the coarser grains picked from the MGS-1 simulant. The file contains a photo of the analyzed crystals (Fig. 3a), the pictures in secondary electrons (SE)taken under the scanning electron microscope of the crystals where the spectra were acquired (Fig. 3b) and the spectrum itself and related elemental ratio from the energy-dispersive spectroscopy analysis (Fig. 3c). The spectra show peaks associated with specific chemical elements, that allow us to understand the chemical composition of the grain. Images and spectra are divided between sections “Dark crystals” and “Red crystals” based on the optical color of the minerals, followed by a short mineralogic interpretation of data reported in Table 5.

**Table 5:** Table of minerals recognized in the crystal analysis of the MGS-1 simulant.

Crystals	Detected minerals	Minor minerals
Black	Pyroxene	Phyllosilicates
Red	Gypsum/Al-oxides	Gypsum, patina of plagioclase or talc

**Fig. 4:**
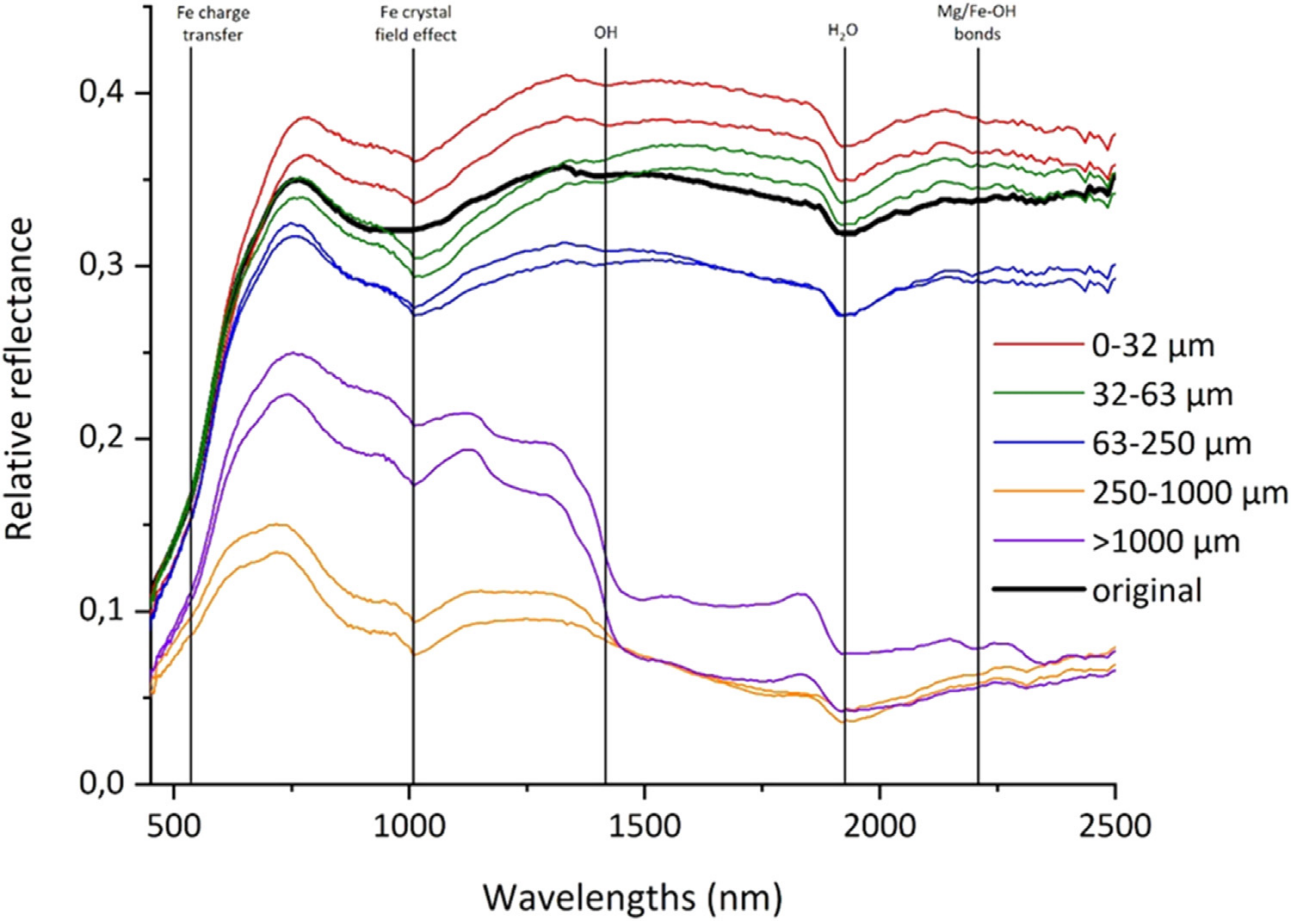
Spectra of the original (i.e., bulk sample) and different grainsize fractions of MGS-1.

The three files in the sub-folder “hyperspectralcameras” have the name of each simulant. For example, in the “MGS-1.docx” file there is the spectral plot (Fig. 4) of the data reported in the Table 6. Thanks to the absorption bands in the graph (Fig. 4) we can identify the main spectral features of the simulant and comprehend the minerals existing within the simulant. The table (Table 6) displays the wavelength range (nm) for the original simulant and for the sieved simulant in the following granulometric classes: 0–32 µm, 32–63 µm, 63–250 µm, 250–1000 µm and >1000 µm. For each class, we acquired two spectra, with the only exception of the >1000 µm-class whose limited amount of material did not allow more than one acquisition (see “MMS-2.docx”). Following Zhang et al. [11], the smoothed/ merged data were multiplied by absolute reflectance of the Spectralon white reference [12] in order to mitigate potential artifacts due to its absorption peak at 2100 nm. Raw data as well as Spectralon white reference data [12] are provided in dedicated tables within the database.

**Table 6:** Extract of the table derived from hyperspectral acquisition of the simulant MGS-1.

Wavelengths (nm)	original	Wavelengths (nm)	0–32 µm		32–63 µm		63–250 µm		250–1000 µm		>1000 µm	
419,26	0,09,186	399,35	0,19,066	0,20,192	0,2148	0,20,697	0,1787	0,18,712	0,15,049	0,14,403	0,1631	0,16,578
421,48	0,08,853	401,56	0,16,198	0,17,347	0,17,115	0,17,426	0,14,673	0,16,301	0,12,402	0,12,088	0,14,095	0,14,537
423,69	0,08,909	403,77	0,14,084	0,15,737	0,14,392	0,15,231	0,1307	0,14,349	0,1097	0,10,655	0,12,311	0,1309
425,90	0,09,141	405,99	0,13,605	0,14,231	0,13,021	0,13,888	0,12,657	0,13,156	0,09,999	0,09,902	0,1098	0,11,735
428,12	0,09,344	408,20	0,13,132	0,13,272	0,12,441	0,1324	0,12,179	0,12,674	0,08,617	0,0916	0,10,067	0,1044
430,33	0,09,355	410,41	0,11,696	0,12,648	0,11,962	0,1236	0,10,825	0,11,653	0,07,239	0,08,222	0,0912	0,08,962
432,54	0,09,383	412,62	0,10,121	0,12,007	0,11,027	0,11,308	0,09,626	0,10,494	0,06,502	0,07,747	0,08,026	0,07,563
434,76	0,09,592	414,84	0,09,332	0,11,169	0,09,536	0,10,107	0,08,837	0,09,409	0,05,733	0,07,066	0,06,903	0,06,658
436,97	0,09,871	417,05	0,08,892	0,10,143	0,08,453	0,092	0,07,904	0,08,394	0,05,078	0,05,754	0,06,016	0,0617
439,18	0,10,076	419,26	0,08,537	0,09,484	0,08,503	0,09,083	0,07,507	0,08,072	0,04,917	0,04,982	0,05,665	0,05,955
441,40	0,10,278	421,48	0,08,549	0,09,402	0,08,549	0,09,131	0,07,552	0,08,188	0,04,881	0,05,102	0,0562	0,0585
443,61	0,10,485	423,69	0,08,558	0,09,443	0,08,331	0,08,887	0,07,544	0,08,195	0,04,832	0,05,189	0,05,616	0,05,628
445,82	0,10,689	425,90	0,08,549	0,09,122	0,0857	0,08,628	0,07,667	0,07,946	0,04,741	0,04,897	0,05,696	0,05,384
448,04	0,10,882	428,12	0,08,563	0,08,981	0,0893	0,08,565	0,07,862	0,07,744	0,0457	0,04,798	0,05,746	0,05,346
450,25	0,11,055	430,33	0,08,466	0,09,015	0,0894	0,08,466	0,07,803	0,07,405	0,04,136	0,04,619	0,05,635	0,05,212
452,46	0,11,189	432,54	0,08,439	0,09,109	0,08,887	0,08,634	0,0768	0,07,267	0,03,957	0,04,398	0,05,536	0,05,072
454,67	0,11,312	434,76	0,08,559	0,09,616	0,08,977	0,09,165	0,07,987	0,07,762	0,0435	0,04,721	0,05,467	0,05,227
456,89	0,1149	436,97	0,08,739	0,10,155	0,0905	0,09,601	0,08,396	0,08,278	0,0469	0,05,144	0,05,505	0,05,509
459,10	0,11,661	439,18	0,08,812	0,10,189	0,09,064	0,09,587	0,08,381	0,08,463	0,04,677	0,0523	0,0563	0,05,688

## 4 Experimental Design, Materials and Methods

### 4.1 Granulometric data

The granulometric analysis has been conducted by means of Laser Diffraction Particle Size Analyzer (Malvern Panalytical Mastersizer3000) equipped with a large volume liquid dispenser (Hydro LV). Samples have been added until a 4 % of light obscuration. Both during the sample addition and the analysis, ultrasound dispersion was activated at the power of 90 %, and the stirring speed has been set to 2500 rpm. In the table below are reported the main parameter for the data acquisition and elaboration (Table 7).

**Table 7:** Main parameters of the Mastersizer3000 software set for this acquisition.

Parameter	Value
Particle shape	Not-spherical
Material	Silica
Refractive index	1.46
Adsorption	0.01
Density (g/cm^3^)	1
Medium	Water
Background	Only water
Blue light	Yes
Number of acquisitions	20
Interval between acquisitions	2 s
Acquisition time	10 s
Obscuration	3–15 %
Shaking	3/4
Ultra-sound	Yes
Cleaning cycle	Automatic
Data processing	General purpose
Grainsize limits	0.1–2000 µm
Results	% volume
Grainsize classes	Udden-Wentworth

Firstly, we have measured the particle size and size distribution of the original simulants (i.e., the bulk samples). After this first screening, the samples have been sieved with an automated vibrating sieve (Retsch) using the following sieve-ranges: 〈32 µm, 32–63 µm, 63–250 µm, 250–1000 µm, 〉 1000 µm. Each fraction has been characterized through the laser diffraction granulometry. For each acquisition, the last five measurements (of 20) have been averaged and reported in a .jpg file, .txt file, and collected in a .docx file.

### 4.2 Chemical data (ICP-MS)

The Inductively Coupled Plasma Mass Spectroscopy Perkin-Elmer NexION 350X has been employed for the quantitative analysis of inorganics elements of the simulants. Around 10 mg of sample has been weighted (Practum, Sartorius, 0.01 mg) and inserted in Teflon vessel for the acidic mineralization assisted by microwave (Ethos UP, Milestone). A mixture of 5 ml of Acqua Regia (3:1 HCl:HNO_3_) and 1 ml of HF has been added to the vessel, and the container has been sealed and microwave heated following the ramp reported in Table 8.

**Table 8:** Parameters for the microwave used for the preparation of ICP-MS samples.

Time (s)	15	20
Temperature (°C)	210	210
Cooling	40 min	
Program name	BCS 300 (Soil)	

The digested has been diluted to a final volume of 50 ml with ultrapure water and analyzed with ICP-MS Perkin Elmer Nexion 350X coupled with the autosampler seaFAST (Direct mode, 4 ml, x10). The selected elements reported in Table 9 have been quantified by means an external eleven-point calibration curves that is also reported in Table 9. The instrumental drift has been corrected by using the Rh (103) as internal standard.

**Table 9:** Elements selected for the chemical analysis and relative parameters used during the acquisition.

Elements	Mass	Mode	He flux (ml/min)	Calibration range (µg/L)
Al	27	KED	4.4	100–1000
Na	23		4.4	
Mg	24		4.4	
Ti	49		4.4	
K	39		4.4	
Ca	43		4.4	
Fe	57		4.4	
Cr	52		4.4	0.5–100
V	51		4.4	
Co	59		0.1	
Ni	60		4.4	
Mn	55		4.4	
Sr	88		4.4	
Cd	111		4.4	
Cu	63		4.4	
Zn	68		4.4	
Ba	137		4.4	
Be	9		0.1	
Pb	208		0.1	
Tl	205		0.1	

For the quality control, “NIST 2711a Montana Soil 2” has been used as Certificate Reference Material (CRM) in order to evaluate the accuracy of the method. The output data are in .txt format as table and converted to a .docx file.

### 4.3 Mineralogical data (X-ray diffraction)

The X-ray diffractometer Philips X'Pert PRO (Bragg-Brentano HD optics, cobalt source, detector X'Celerator detector) has been used for mineralogical characterization of the three Martian simulants. We have prepared the samples in different ways in function of the analysis to be carried out as follows:

- Qualitative analysis: manual grinding of dry simulants with agate mortar (<63 µm).
- Quantitative analysis – Rietveld method: the samples was weighted and mixed with an appropriate amount of standard zincite ZnO in order to be 20 % of the whole sample. A specific amount of ethanol was added to the mixture of MMS-1 + zincite and of MMS-2 + zincite, which was put in the micronizer at highest speed for 5 min. The powder was dried in air. The mixture MGS-1 + zincite was gridded by a dry bead mill because of the solubility of the Mg-sulphate in MGS-1The result was a uniform and fine powder (<5 µm) ready to be analyzed.

HighScore (Plus) software version 4.9 (PANalytical B.V., 2020, Almelo, The Netherlands) [13] allows to identify the mineral species (qualitative phase analysis) while the relative abundance of each phase has been calculated using the Rietveld method as implemented in Profex-BGMN v. 5.2.3 [14]. The plots and the table with minerals and relative amounts are exported as .jpg file and .txt file, respectively, and collected in a .docx file. The following Table 10 summarizes all the information.

**Table 10:** Main parameters about the XRD acquisitions.

Instrument	Philips X'Pert Pro Diffractometer (Almelo, The Netherlands) working with parafocusing Bragg Brentano geometry
X-ray tube	Long Fine Focus, Co source, 40 kV and 40 mA generator settings
Detector	Real-Time Multiple Strip (RTMS) X'Celerator
Sample holder	Circular, 27 mm diameter and 2 mm thick. Back-loading filling
Accessories	Bragg-BrentanoHD ©, spinner for sample revolution on vertical axis
Slits	Divergence slits 1/4°, antiscatter slits 1°, Soller silits 0.04 rad
Data acquisition	3–85° 2theta, continous scan lasting 1 h (100 s for 0.017° 2theta virtual steps).
Micronization procedure	5 min grinding using ethanol at 1500 rpm/min. Instrument: Retsch XRD-MILL MCCRONE.
Dry grinding procedure	3 min dry grinding at 20 Hz frequency. Instrument: Retsch MM 400
Qualitative phase analysis	HighScore (Plus) software version 4.9, year 2020, by PANalytical B.V., Almelo, The Netherlands [13]
Quantitative phase analysis	Profex-BGMN v.5.2.3 [14]

### 4.4 Chemical data of MGS-1 (SEM-EDS)

Chemical analyses have been acquired with the dual beam scanning electron microscope FEG-FIB Tescan SOLARIS. The instrument is equipped with the Ultim Max 65 Silicon Drift EDS made by Oxford Instruments. This detector was used to confirm the chemical composition of the mineralogical phases in the MGS-1 coarse black and reddish grains. We worked at 15 KeV, 3 nA with a working distance of 5 mm. The crystals have been coated with Chromium before the analyses to prevent charging. Since only semi-quantitative data were acquired, the samples were not polished and no standardization was needed, we only performed the current calibration of the cobalt standard and then we used an internal standardization in the Aztec software made by Oxford Instruments. The output are highly-detailed photos, in which sites of chemical acquisitions are displayed with the corresponding spectra where each peak is representative of a specific element, from which to infer the mineralogy. Images and diagrams are exported as .jpg file and collected in a .docx file.

### 4.5 Hyperspectral data

The hyperspectral imaging cameras used are:

• Headwall Photonics Nano-Hyperspec: push-broom camera, spectral range from 400 to 1000 nm, 270 spectral bands, 640 spatial bands, sampling of ∼2 nm, spatial resolution varying with the height of the camera in its stage;
• Headwall Photonics Micro-Hyperspec camera: push-broom camera, spectral range from 900 to 2500 nm, 170 spectral bands, 384 spatial bands, sampling of ∼10 nm, spatial resolution varying with the height of the camera in its stage.

The laboratory setup accommodation for hyperspectral acquisitions consists in: a camera holder and a motorized stage of 20 × 20 cm in dimension and capable to sustain up to 20 kg while illumination is equipped by a tiltable halogen lamp provided with a ground glass diffuser. The whole system measures 100 × 45 × 78 cm. After setting up parameters in the camera software, original simulants were placed on the motorized stage to acquire their spectra both in VNIR and SWIR range (Table 11). As white reference for radiance conversion a 99 % reflectivity Spectralon (Labsphere, Inc.) [12] was used, we repeated the acquisition twice with each grainsize-class of the three simulants.

**Table 11:** Main parameters of the Headwall photonics nano-hyperspec and micro-hyperspec for this acquisition.

Camera	VNIR	SWIR
Exposure (ms)	14	10
Frame period (ms)	25	15
Lens EFL (mm)	17	25
Array Pixel Pitch (µm)	7.4	24
Camera FPS	40.13	61.84
Write FPS	40	60
Stage speed (mm/s)	9.1	18
Height (cm)	52.5	28
Spatial pixel size (mm)	∼0.25	∼0.25

Acquisition output files were imported in ENVI software [15] where Regions of Interest (ROIs) were defined and spectral data extrapolated. The obtained files in ASCII format were opened and processed in Origin software [16]. A light smoothing (binomial method) was applied in the VNIR spectra to reduce signal noise and finally VNIR and SWIR acquisitions were merged at 970 nm. For the sieved simulants, merging consisted in a downward shift of the SWIR curve, while for the original VNIR and SWIR simulants values around the merging point were averaged. Therefore, the original and sieved samples have different x-axis values in the merging region. Moreover, the smoothed/merged data were multiplied by absolute reflectance of the Spectralon white reference [12] to mitigate potentials artifacts [11]. Finally, plots and tables of the spectral acquisitions were exported as a jpg. file and .txt file and collected in a .docx file.

## Limitations

Global and commercially available Martian simulants are still few and, among the most accessible ones, MMS-1 and MMS-2 are only partially representative of the global dust [2]. This raises the need to identify or eventually create through appropriate mixtures new and even more realistic natural simulants. Although X-ray diffraction is a very efficient technique, overlapping peaks might leave some margin to different interpretations. For this reason we have provided even the diffraction patterns in a numerical format. The EDS results do not show any peak overlapping being detected only well distinguishable pyroxenes, feldspars and sulphates on major grains of MGS-1 whose composition was already detected through the XRD analysis.

## Ethics Statement

All authors have read and followed the ethical requirements for publication in Data in Brief and confirming that the current work has not involved human subjects, animal experiments, or any data collected from social media platforms.

## Credit Author Statement

**Nicole Costa:** Conceptualization, Validation, Formal analysis, Investigation, Data Curation, Writing – Original Draft, Writing – Review & Editing, **Alessandro Bonetto:** Methodology, Validation, Formal analysis, Investigation, Resources, Data Curation, Writing – Review & Editing, **Patrizia Ferretti:** Resources, Supervision, Writing – Review & Editing, **Bruno Casarotto:** Methodology, Validation, Formal analysis, Investigation, Data Curation, Writing – Review & Editing, **Matteo Massironi:** Resources, Writing – Review & Editing, Supervision, **Francesca Altieri:** Supervision, Writing – Review & Editing, **Jacopo Nava:** Validation, Formal analysis, Investigation, Data Curation, **Marco Favero:** Validation, Formal analysis, Investigation, Data Curation.

## Data Availability

Research Data UnipdMartian simulant analysis dataset (Original data)
